# Ruthenium-106 Plaque Therapy for Diffuse Choroidal Hemangioma in Sturge-Weber Syndrome

**DOI:** 10.1155/2011/785686

**Published:** 2011-09-29

**Authors:** Agnieszka Kubicka-Trząska, Joanna Kobylarz, Bożena Romanowska-Dixon

**Affiliations:** Department of Ophthalmology and Ocular Oncology, Medical College, Jagiellonian University, Kopernika Street 38, 31-501 Kraków, Poland

## Abstract

Diffuse choroidal hemangiomas associated with Sturge-Weber syndrome (SWS) are classically treated with external beam radiotherapy (EBR), but there are a few reports usually of single cases indicating the usefulness of plaque therapy. We present our observations on two cases of diffuse choroidal hemangiomas with exudative retinal detachment associated with SWS treated with Ruthenium-106 plaque therapy. Outcomes included best-corrected visual acuity (BCVA) and regression in tumor thickness measured by ultrasonography. The initial BCVA of the affected eyes was counting fingers at 1 meter and light projection. Pretreatment tumors thickness was 3.5 mm and 4.7 mm. In a follow-up period of 18–24 months, significant reduction in thickness of choroidal hemangiomas up to 1.2 mm and 1.4 mm with prompt resolution of exudative retinal detachment was observed. BCVA achieved 20/200 and 20/400, respectively. The findings in this paper indicate that Ruthenium-106 plaque therapy is effective in treatment of diffuse choroidal hemangiomas associated with SWS.

## 1. Introduction

 The Sturge-Weber syndrome (SWS), also called encephalotrigeminal angiomatosis, is a sporadically occurring neurocutaneous disorder with capillary venous angiomas involving the skin of the face, typically in the ophthalmic (V1) and maxillary (V2) distributions of the trigeminal nerve, choroid, and leptomeninges [1]. Choroidal hemangiomas are estimated to occur in approximately one third of SWS cases, and they may occur in childhood or in early adulthood [2]. Choroidal hemangiomas are usually diffuse, primarily related to the posterior pole, and often seen as orange-red, flat to moderately elevated masses. Diffuse choroidal hemangiomas may be associated with an exudative retinal detachment with macular involvement, photoreceptor cell loss and cystoid degeneration of the sensory retina causing visual loss [3]. These benign tumors are classically treated with external beam radiotherapy (EBR) [4, 5]. There are also reports on single cases indicating the usefulness of plaque therapy [6, 7]. 

We present our observations on two cases of diffuse choroidal hemangiomas with exudative retinal detachment associated with SWS treated with Ruthenium-106 plaque therapy. 

## 2. Report of Cases

### 2.1. Case  1

**Figure 1:**
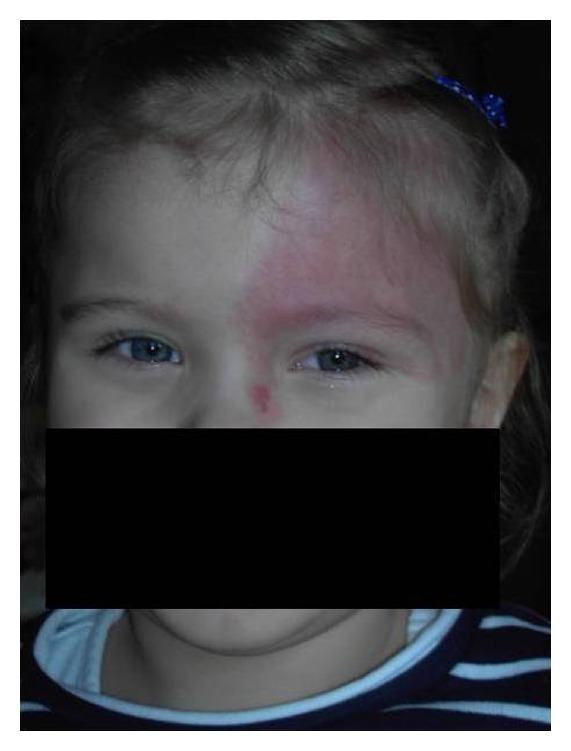
Diffuse nevus flammeus involving the left upper eyelid and forehead associated with Sturge-Weber syndrome.

**Figure 2:**
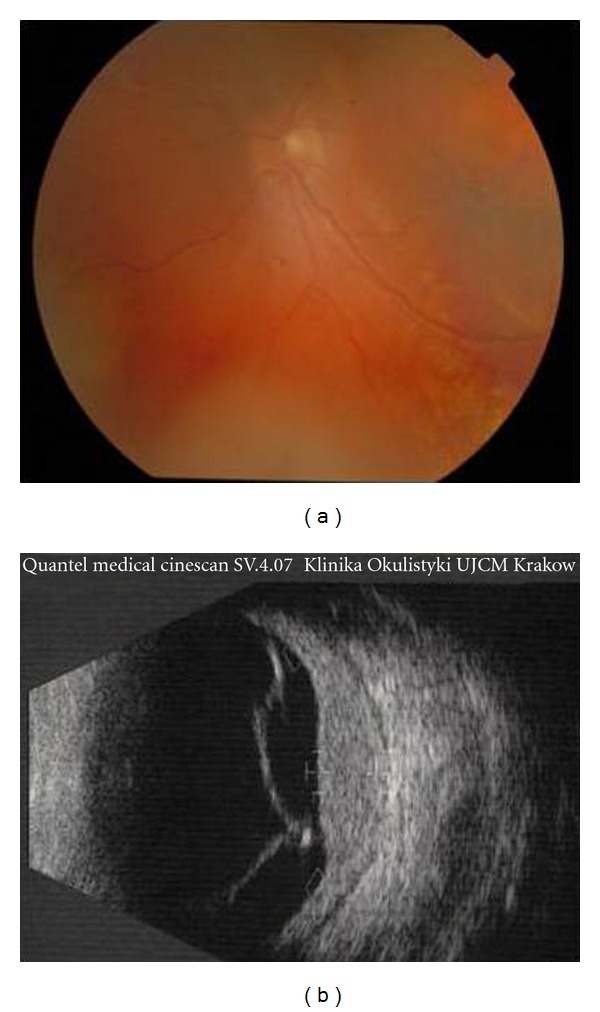
Fundus and B-scan ultrasonography demonstrating diffuse choroidal hemangioma with extensive exudative retinal detachment.

A 6-year-old girl presented with blurred vision in the left eye of 1-month duration. On examination the best corrected visual acuity (BCVA) was 20/20 OS and counting fingers at 1 meter OD. She had diffuse nevus flammeus involving the left upper eyelid, forehead, and bulbar conjunctiva suggestive of Sturge-Weber syndrome (Figure 1). No other pathology was found on slit-lamp examination. The intraocular pressure was within normal limits. The fundus of the left eye showed a diffuse red-orange choroidal lesion involving the posterior pole and temporal part of the fundus with exudative retinal detachment (Figure 2(a)). B-scan ultrasonography demonstrated a diffuse choroidal mass 3.5 mm in thickness and 15.8 mm in diameter with regular high internal reflectivity typical for choroidal hemangioma (Figure 2(b)). The anterior segment and fundus of the right eye were normal. Computed tomography (CT) scans of the brain were unremarkable. Nuclear magnetic resonance (NMR) images showed the presence of a pineal gland cyst. A Ruthenium-106 plaque (CCB, Bebig Isotopen, Germany) was used which delivered a dose of 30 Gy to the tumor apex. 

**Figure 3:**
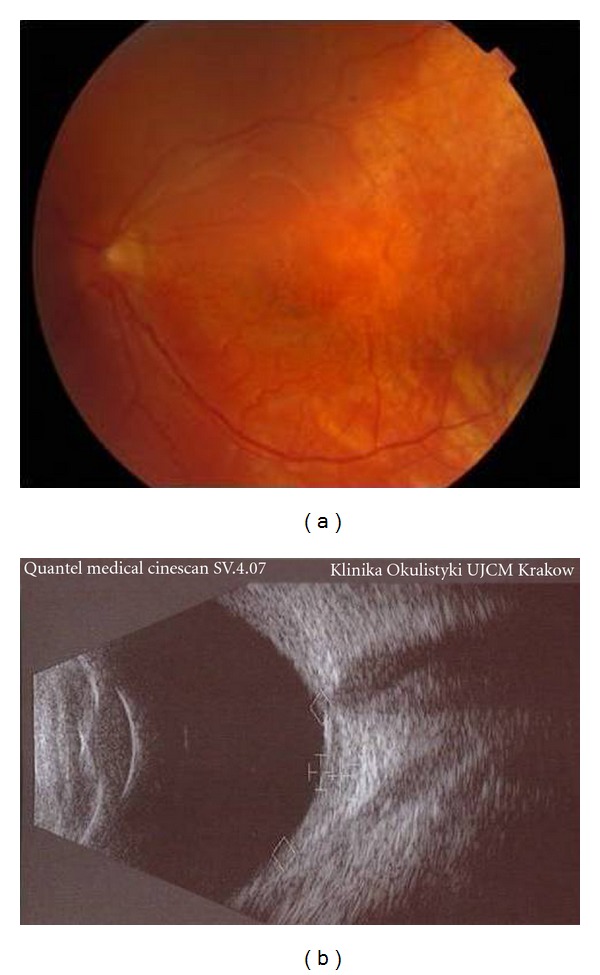
The same eye 18 months after Ruthenium-106 plaque therapy—regression of the tumor, no subretinal fluid is present.

At 1-month posttreatment, the thickness of the hemangioma had decreased to 2.1 mm with partial resorption of subretinal fluid and BCVA was 20/400. Three months after therapy, tumor thickness was 1.4 mm, no retinal detachment was observed and BCVA improved to 20/200. Six months after treatment, BCVA was stable and the hemangioma was nearly flat (1.2 mm) (Figures 3(a) and 3(b)). Next follow-up visits performed at the 12th and 18th months after plaque therapy revealed further stabilization of both functional and anatomical outcomes. No radiation-induced complications were observed.

### 2.2. Case  2

A 6-year-old girl presented with diffuse bilateral nevus flammeus involving the upper eyelids, forehead, and elevated intraocular pressure in both eyes. Anamnesis revealed episodes with epilepsy, which had started at the age of 5 months. On examination, BCVA was 20/20 OD and light projection OS. Anterior segment examination showed the presence of conjunctival hemangioma in the left eye. The intraocular pressure was 18 mmHg OD and 17 mmHg OS. Fundoscopy of the left eye showed total exudative retinal detachment. The fundus of the right eye was normal. A- and B-scans ultrasonography of the left eye revealed diffuse thickening of the choroid suggestive of diffuse choroidal hemangioma and extensive exudative retinal detachment. The tumor thickness was 4.7 mm, with the largest diameter of 15.6 mm. CT scans and NMR images of the brain showed the presence of diffuse left cerebral angiomas. A Ruthenium-106 plaque (CCB, Bebig Isotopen, Germany) was used and delivered a dose of 30 Gy to the tumor apex.

 At 1-month posttreatment, the maximum thickness of the choroidal hemangioma had decreased to 2.8 mm with partial subretinal fluid resorption, BCVA was counting fingers at 1 meter OS. The followups performed 3 and 6 months after therapy showed decreased tumor thickness to 2.4 and 2.0 mm, respectively. Six months after treatment, no serous retinal detachment was present and BCVA was counting fingers at 3 m OS. After 12 months, the tumor thickness measured 1.4 mm and BCVA improved to 20/400. Twenty-four months after treatment, tumor thickness was unchanged and visual acuity remained stable. No radiation-induced complications were noted.

## 3. Discussion

EBR has been recommended for diffuse choroidal hemangiomas with the presence of exudative retinal detachment [4, 5]. However, this therapy is associated with slow absorption of subretinal fluid, usually taking months. Recurrence or persistence of the exudative retinal detachment is often observed, which makes additional EBR necessary with the risk of cataract, radiation retinopathy, and neuropathy development [5]. 

Brachytherapy with radon seeds for the treatment of circumscribed choroidal hemangioma was first described by MacLean and Maumenee in 1960 [8]. Plaque radiation therapy with Cobalt-60, Palladium-103, Iodine-125, and Ruthenium-106 is considered an effective and safe method of therapy for large circumscribed choroidal hemangiomas with subretinal fluid [6, 7, 9, 10]. The literature describes only three cases of diffuse choroidal hemangiomas treated with plaque radiation therapy. Zografos et al. [6] used Cobalt-60 applicators in two patients and Murthy et al. [7] used a Ruthenium-106 plaque in one case of diffuse choroidal hemangioma with exudative retinal detachment associated with SWS. They observed early and complete resolution of the subretinal fluid and tumor regression with improvement in BCVA in treated eyes. In our patients, the subretinal fluid was completely resolved within 3–6 months and the choroidal hemangiomas showed significant size reduction as observed on B-scan ultrasonography. Both patients demonstrated improvement in BCVA. 

Our observations indicate that plaque therapy is an effective and safe treatment option for diffuse choroidal hemangiomas associated with SWS.

##  Conflict of Interest

The authors have no financial interest that is related to the manuscript.
